# Age-Friendly Initiatives and Primary Care in Rural Arkansas

**DOI:** 10.1093/geroni/igaa057.1778

**Published:** 2020-12-16

**Authors:** Robin McAtee, Leah Tobey

**Affiliations:** 1 UAMS, Little Rock, Arkansas, United States; 2 University of Arkansas for Medical Sciences, Little Rock, Arkansas, United States

## Abstract

The Arkansas Geriatric Education Collaborative (AGEC)’s Geriatric Workforce Enhancement Program is partnering with a plethora of community based organizations (CBO) and with ARcare, an Arkansas federally qualified healthcare clinic network, to implement the 4Ms of age-friendly care in rural clinics. Baseline clinical data related to the Age-Friendly 4M Framework has been gathered and quality improvement projects initiated to improve the outcomes. Initiatives to improve depression and cognitive screenings are addressing Mentation; fall prevention screens and the offering of fall prevention programs have been added for Mobility; high risk medication screens and chronic pain educational programs are being implemented to address Medications; and finally, Medicare Annual Wellness Visits is the cornerstone to improve what Matters to older adults. A campaign that involves partnered CBOs to address health literacy and increase involvement in evidence-based programs is also helping to drive improvements in age-friendly care in rural Arkansas.

